# The regulatory mechanisms and clinical translation potential of RNA-binding protein RALY in tumors

**DOI:** 10.3389/fonc.2026.1806422

**Published:** 2026-04-22

**Authors:** Jiale Huang, Lizhou Jia, Yuexin Liu, Lingna Gao, Zhihui Weng, Yanmei Li

**Affiliations:** 1 Department of Gastroenterology, Affiliated Hospital of Inner Mongolia Medical University, Hohhot, Inner Mongolia, China; 2 Central Laboratory, Bayannur City Hospital, Bayannur, Inner Mongolia, China

**Keywords:** hnRNP, metabolic reprogramming, regulatory mechanisms, RNA-binding protein RALY, therapeutic target, tumor

## Abstract

The RNA-binding protein RALY is an important member of the heterogeneous nuclear ribonucleoproteins (hnRNPs). It can target and regulate the key links of the RNA metabolic networks, such as RNA alternative splicing and stability maintenance, and is deeply involved in biological processes such as cell proliferation, metabolic reprogramming, and exosome biogenesis. RALY functions as a core regulator in tumorigenesis and tumor progression. In recent years, studies on RALY in cancer have been increasing. It is abnormally highly expressed in hepatocellular carcinoma, colorectal cancer, lung cancer, and other tumors. Emerging evidence suggests its oncogenic functions, multiple regulatory mechanisms, and potential clinical translational value, providing a candidate target for tumor precision therapy. At the mechanism level, RALY mainly plays a role in promoting cancer by regulating the three core methods of target gene alternative splicing, post-translational modifications (PTMs) of itself (ubiquitination, glycosylation, etc.), and mediating tumor metabolic reprogramming, thereby driving malignant biological behaviors such as tumor cell proliferation, invasion, metastasis, and chemotherapy resistance. At the clinical level, high RALY expression is associated with poor patient prognosis, indicating that it may serve as a promising candidate prognostic marker. This article reviews the molecular structure and core functions of RALY, focuses on its regulatory mechanisms and clinical significance in various tumors, and discusses the prospects and challenges of targeting RALY so as to provide theoretical support and direction for basic research and clinical translation of RALY-related tumors.

## 1 Introduction

HnRNPs constitute a highly conserved class of RNA-binding proteins (RBPs) and represent one of the most abundant protein families in the nucleus. They are involved in almost the entire gene expression process from RNA transcription to protein synthesis (1, 2). The hnRNP family comprises more than 30 members, with molecular weights ranging from 34 to 120 kDa. Members are named in alphabetical order (from hnRNP A1 to hnRNP U), and there are significant differences in sequence characteristics, subcellular localization, and functional preferences among different members (3, 4). RALY (hnRNP-associated with lethal yellow, also known as HNRPCL2 or P542) is an important member of the hnRNP C family, and the amino acid sequence identity with the family member hnRNP C is as high as 43%. The protein was initially identified as an autoantigen that cross-reacted with Epstein-Barr nuclear antigen 1 (EBNA1). Subsequent studies by Michaud et al. found that an RNA-binding protein (RBP) related to the embryonic lethal phenotype of homozygous lethal yellow mice could regulate the embryonic development of the mice and named it RALY (5). As a member of the hnRNP C family, RALY has the common RNA-binding characteristics of the hnRNP family. Through its RNA recognition motif (RRM) domain, it specifically recognizes and binds to sequence motifs such as poly-uridine (poly-U) of RNA and plays a central role in gene expression regulation. This protein is widely involved in alternative splicing of precursor messenger RNA (pre-mRNA) (6–9), mRNA stability maintenance (10, 11), non-coding RNAs (ncRNAs) regulation (12, 13), as well as transcription (14, 15) and translation (16), thereby regulating important biological processes such as cell proliferation, mitochondrial metabolism, lipid metabolism, and exosome biogenesis.

RALY is widely distributed in human tissues, especially in the nervous system, kidneys, liver, skeletal muscle, lungs, and pancreas (17). Studies have shown that abnormal expression of RALY can interfere with normal physiological functions. It is not only closely related to viral infection (18, 19), metabolic diseases (20), and neuropathic pain (21), but also involved in the development and progression of various malignant tumors (22, 23). In recent years, more and more studies have confirmed that RALY is abnormally highly expressed in multiple tumor tissues, including hepatocellular carcinoma, colorectal cancer, pancreatic cancer, lung cancer, breast cancer, glioma, and cervical cancer. This protein not only plays a key oncogenic role in tumorigenesis, but its overexpression is also associated with poor survival and prognosis of patients (10, 24–27). Therefore, this review aims to summarize the latest research progress on the RNA-binding protein RALY in the digestive system and other systemic tumors. Through in-depth analysis of the structure-function relationships of RALY, its tumor-specific regulatory mechanisms in different tumors, clinical application potential, and targeted intervention strategies. By doing so, it can provide critical theoretical evidence for clarifying the unique biological roles of RALY in tumors and advancing the development of precision oncology.

## 2 Structure and molecular function of RALY

### 2.1 Structural characteristics of RALY

**Figure 1:**
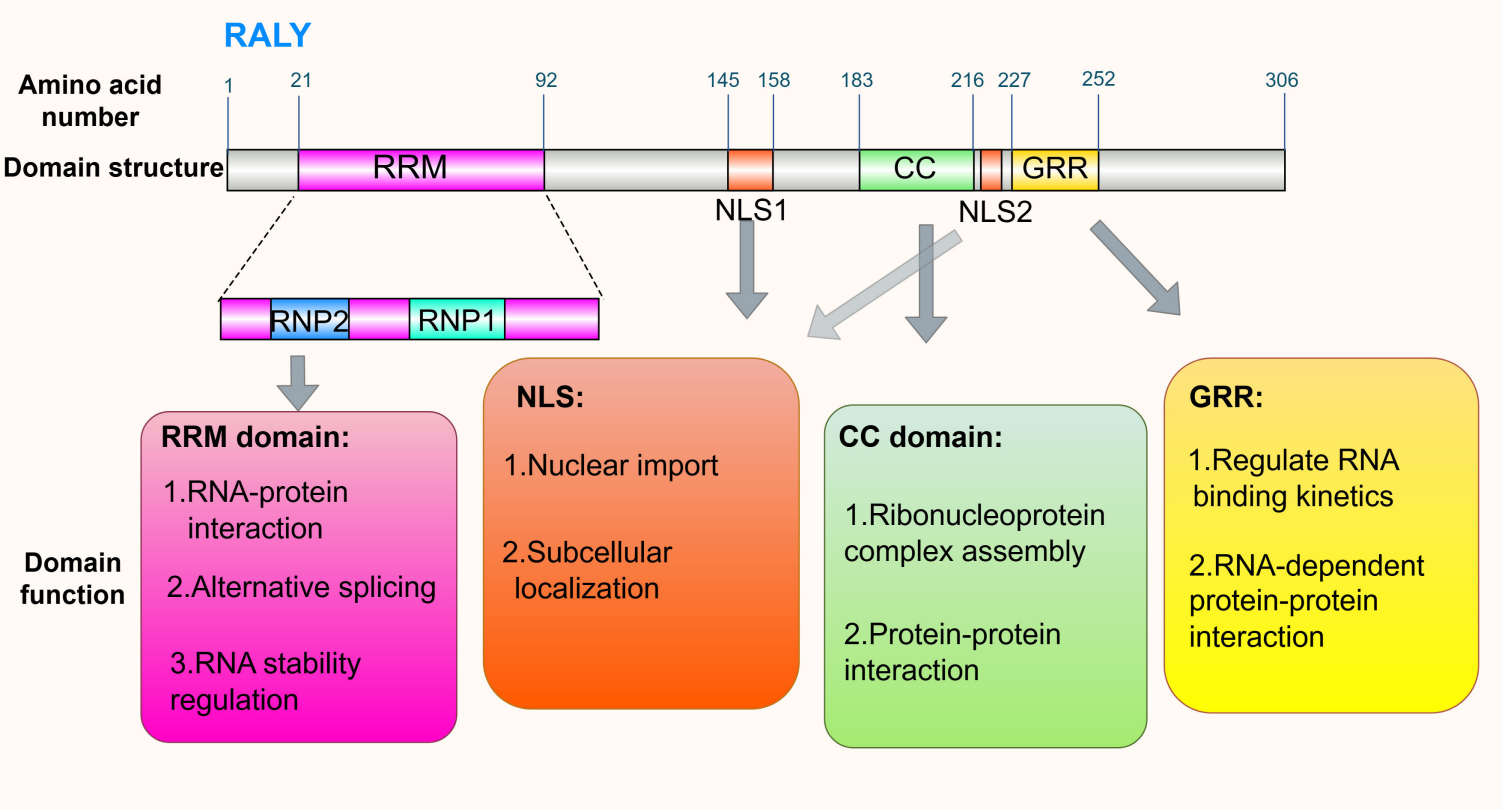
Domain distribution and functions of RALY. Domain distribution: RALY comprises the RRM domain [residues 21-92, containing RNP2 (23-28) and RNP1 motifs (55-62)], NLS1 (residues 145-158), CC domain (residues 183-216), NLS2 (residues 218-224), and GRR domain (residues 227-252). Functions of each domain: RRM domain: Participates in RNA-protein interaction, alternative splicing, and regulation of RNA stability; NLS: Responsible for nuclear import and subcellular localization; CC domain: Involved in ribonucleoprotein complex assembly and protein-protein interaction; GRR: Regulates RNA binding kinetics and RNA-dependent protein-protein interaction. By Figdraw.

The RALY gene is localized to chromosome 20q11.22, encoding a protein of 306 amino acids with a molecular weight of about 36 kDa. It shares 87.5% amino acid sequence identity with the mouse homolog. This protein is mainly localized in the nucleus, with only minor distribution in the cytoplasm (5). The molecular structure is the material basis for the biological function of RALY. Combined with bioinformatics analysis and experimental validation results, it has been clarified that it contains four major core domains, as shown as in **Figure 1** (18, 28). There is a typical RRM in the N-terminal region, which is the core functional element for the specific binding of RALY to RNA. This domain contains two highly conserved RNA-binding motifs, ribonucleoprotein 1 (RNP1) and ribonucleoprotein 2 (RNP2). It can specifically recognize and bind to the poly-U sequences within the 3’ untranslated region (3’UTR) of mRNA, which provides a key molecular basis for regulating the alternative splicing of pre-mRNA and maintaining the stability of mRNA. There is a glycine-rich region (GRR) in the C-terminal region. While the specific function of this domain has not been fully elucidated, existing studies have confirmed that it does not affect the RNA-binding activity of RALY. It is speculated that GRR may exert its biological functions by regulating RNA binding kinetics (e.g., binding stability and dissociation rate) and mediating RNA-dependent protein-protein interactions (PPIs). The middle region contains two independent nuclear localization signals (NLSs, named NLS1 and NLS2, respectively), which are the key components that mediate RALY nuclear localization. Studies have found that mutations in NLS1 or NLS2 can cause RALY to remain in the cytoplasm and form abnormal aggregates, thereby interfering with RNA metabolism, suggesting that the normal function of RALY depends partly on its own nuclear localization characteristics (29). In addition, there is a highly conserved coiled-coil domain (CC) in the central region of the protein molecule. The core function of this domain is to mediate PPIs by hydrophobic interaction and to drive the formation of RALY homodimers and other polymers, thereby providing indispensable structural support for the assembly of ribonucleoprotein (RNP) complexes.

### 2.2 Molecular functions of RALY

As a kind of RBP with multiple biological functions, the function of RALY depends on the specific binding ability to RNA molecules. By regulating the key links of RNA metabolism and the functional activity of ncRNAs, RALY can achieve fine regulation of gene expression, which is embodied in the following three aspects:

#### 2.2.1 Regulation of Pre-mRNA alternative splicing

RALY is part of the spliceosomal C complex, which can be directly involved in the splicing process of various pre-mRNAs based on the RNA binding capacity of the N-terminal RRM. In the HeLa cervical cancer cell line, RALY modulates the expression of immunity and inflammatory response-related genes by regulating the alternative splicing of the transcription factor FBJ osteosarcoma oncogene (FOS), thereby affecting the key biological behaviors of cancer cells, including proliferation, apoptosis, and metabolism (23). In prostate cancer, RALY forms a functional complex with polypyrimidine tract binding protein 1 (PTBP1) through the interaction verified by co-immunoprecipitation, which jointly regulates the splicing switch of DNA methyltransferase 3B (DNMT3B) pre-mRNA, promotes the generation of the oncogenic isoform DNMT3B-L, induces abnormal DNA methylation, and ultimately enhances the radiation resistance of tumor cells (30).

#### 2.2.2 Maintenance of mRNA stability

RALY differentially regulates the stability of target transcripts by specifically binding to the poly-U element of mRNA 3’UTR, thereby affecting the expression level of downstream proteins. It has been confirmed that RALY can bind to the 3’UTR of E2F transcription factor 1 (E2F1) mRNA and significantly enhance its stability, thereby increasing E2F1 protein expression. As a classical factor regulating cell proliferation, the up-regulation of E2F1 expression can directly promote the continuous progress of the cell cycle (10). In addition, RALY can also bind to the mRNA 3’UTR of annexin A1 (ANXA1) and histone H1X (H1FX). Through the selective regulation of the stability of these two transcripts, the expression level of ANXA1 protein is up-regulated and the expression level of H1FX protein is down-regulated (5). It is worth noting that E2F1, ANXA1, and H1FX all play a role in the core biological process of cell proliferation. RALY forms a functional synergy by regulating the expression of the three and constructs its core molecular mechanism of regulating cell proliferation, which provides an important theoretical basis for its participation in tumorigenesis and development.

#### 2.2.3 Non-coding RNAs regulation

The function of RALY is not limited to protein-coding mRNAs; it also expands the gene regulatory network through specific interactions with ncRNAs and participates in the regulation of multiple physiological and pathological processes (**Table 1**): in the field of lipid metabolism, RALY can interact with the long non-coding RNA (lncRNA) LeXis in mouse liver and the long intergenic non-coding RNA regulating sterol homeostasis (lincNORS) in hormone-responsive tissues, thereby participating in the maintenance of systemic sterol homeostasis via regulating the functions of these ncRNAs (12, 31). In the regulation of cell proliferation, RALY is the core dependent factor for lncRNA E2F1 mRNA stabilizing factor (EMS) to exert carcinogenic effects: RALY can specifically bind to the poly-U sequence of lncRNA EMS, thereby enhancing the regulation of EMS on the stability of E2F1 mRNA and the promotion of cell proliferation, and ultimately promoting tumorigenesis. On the contrary, knockdown of RALY can completely block the cancer-promoting effect of EMS (32). In telomere homeostasis regulation, RALY can directly bind to the UUAGGG repeat sequence at the 3’ end of non-polyadenylation telomere-related lncRNA (TERRA) through its RRM domain so as to maintain the structural stability of the TERRA molecule, thereby ensuring the integrity of telomere structure and ultimately avoiding the abnormal activation of the DNA damage response pathway (13). Meanwhile, the function of RALY is also regulated by ncRNAs. In hepatoblastoma, RALY is a downstream target gene of the lncRNA ZNFX1 antisense RNA 1 (ZFAS1)/miR-193a-3p regulatory axis; as a competitive endogenous RNA (ceRNA), ZFAS1 removes its inhibitory effect on RALY by adsorbing miR-193a-3p, thereby activating the RALY/hepatocyte growth factor (HGF)/mesenchymal-epithelial transition factor (c-MET) signaling axis, which has been shown to be a key driver of tumor epithelial-mesenchymal transition (EMT) and invasion and metastasis (33–35). In the regulation of HIV infection, RALY can bind to the 3’UTR region of C-C chemokine receptor type 5 (CCR5) mRNA and promote its degradation, thereby limiting HIV infection. In contrast, CCR5 antisense (CCR5AS) lncRNA can be used as a “molecular decoy” for RALY to relieve its inhibitory effect on HIV infection by preventing the binding of RALY to CCR5 mRNA and inhibiting the function of RALY (36). In summary, a bidirectional regulatory interaction mode exists between RALY and ncRNAs. RALY can regulate the functions of various ncRNAs through specific binding, thereby participating in the regulation of multiple physiological and pathological processes such as lipid metabolism, cell proliferation, and telomere homeostasis. Meanwhile, its own expression and function can be targeted and regulated by ncRNAs, which in turn allows it to participate in the regulation of tumor-related signaling pathways and viral infection and other processes. However, current research on the interaction between RALY and ncRNAs is limited to a small number of molecules. Future studies need to further expand the research scope, explore the regulatory effects of RALY on more types of ncRNAs, and conduct an in-depth analysis of the latest research findings related to its downstream functional regulatory network.

**Table 1:** Bidirectional regulation between RALY and ncRNAs.

Regulatory direction	Type of ncRNA	Mechanism	Biological effect	References
RALY regulating ncRNAs	LincNORS, lncRNA LeXis	Specifically binds and modulates their functions	Maintains sterol homeostasis and regulates lipid metabolism	(12, 31)
RALY regulating ncRNAs	lncRNA EMS	Binds its poly-U sequence to enhance EMS-mediated E2F1 mRNA stability	Promotes cell proliferation and tumorigenesis	(32)
RALY regulating ncRNAs	lncRNA TERRA	Binds its 3’ UUAGGG repeats to maintain TERRA stability	Preserves telomere integrity and inhibits abnormal DNA damage response	(13)
ncRNAs regulating RALY	lncRNA ZFAS1/miR-193a-3p	ZFAS1 adsorbs miR-193a-3p, activates RALY/HGF/c-MET axis	Mediates tumor EMT and invasion	(33)
ncRNAs regulating RALY	lncRNA CCR5AS	Acts as molecular decoy to block RALY-CCR5 mRNA binding	Abrogates RALY’s inhibition of HIV infection	(36)

## 3 Regulatory mechanisms and networks of RALY

### 3.1 Regulation of RALY by m^6^A epigenetic modification

N6-methyladenosine (m^6^A) is the most common epigenetic modification of RNA in cells, which can achieve reversible regulation of RNA function, thereby affecting the processing and metabolism of RNA in various diseases, especially in tumors. m^6^A-dependent epigenetic regulation represents one of the key mechanisms governing RALY function (37, 38). Similar to most hnRNP family proteins (e.g., hnRNP C and hnRNP A2B1) (39, 40), RALY participates in RNA processing and maturation through an “m^6^A-switch” mechanism. This mechanism entails that m^6^A modification alters the secondary structure of target RNAs, thereby influencing the interaction between RBPs and their target RNAs (41). In colorectal cancer, the mechanism is as follows: RALY regulates the post-transcriptional processing of microRNAs (miRNAs) such as miR-483, miR-676, and miR-877 in an m^6^A-dependent manner. Colorectal cancer can progress more quickly as a result of these mature miRNAs’ ability to further mediate the reprogramming of mitochondrial metabolism in tumor cells (42). In conclusion, RALY uses the “m^6^A-switch” mechanism to indirectly regulate primary microRNA (pri-miRNA) processing. This m^6^A-dependent regulatory pattern clarifies RALY’s functional role within the epigenetic modification network. It also reveals a novel epigenetic pathway that enables RALY to participate in tumor metabolic reprogramming.

### 3.2 Protein-protein interactions regulate RALY function

PPIs represent another critical pathway for RALY to exert its biological functions. RALY can form complexes with proteins of different functions to achieve functional expansion and precise regulation. Tenzer et al. used proteomic analysis techniques and identified 143 high-confidence RALY-interacting proteins under physiological conditions. These proteins include RBPs such as hnRNPs and serine/arginine-rich (SR) proteins, as well as translation-related regulatory factors. These proteins specifically form RNPs with RALY, which are mainly involved in mRNA splicing, transport, stability maintenance, and translation regulation (17). Furthermore, RALY can jointly regulate downstream signaling pathways by forming specific complexes with transcription factors, splicing factors, and other proteins (43). In one instance, RALY forms a complex with nuclear factor Y (NF-Y) in the regulation of the cholesterol synthesis pathway, binds to the promoter region of sterol regulatory element-binding protein 2 (SREBP2), dramatically increases its transcriptional activity, and then selectively controls the expression of important genes involved in cholesterol synthesis (20). RALY suppresses the p53-mediated tumor-suppressive pathway in lung cancer by forming a ternary complex with murine double minute 2 (MDM2) and ubiquitin-specific protease 7 (USP7) (44). As a result, RALY widely participates in a range of physiological and pathological processes, including the development of lung cancer and the metabolism of cholesterol, via the formation of RNP complexes or signal transduction complexes.

### 3.3 Dynamic regulation of post-translational modifications

**Figure 2:**
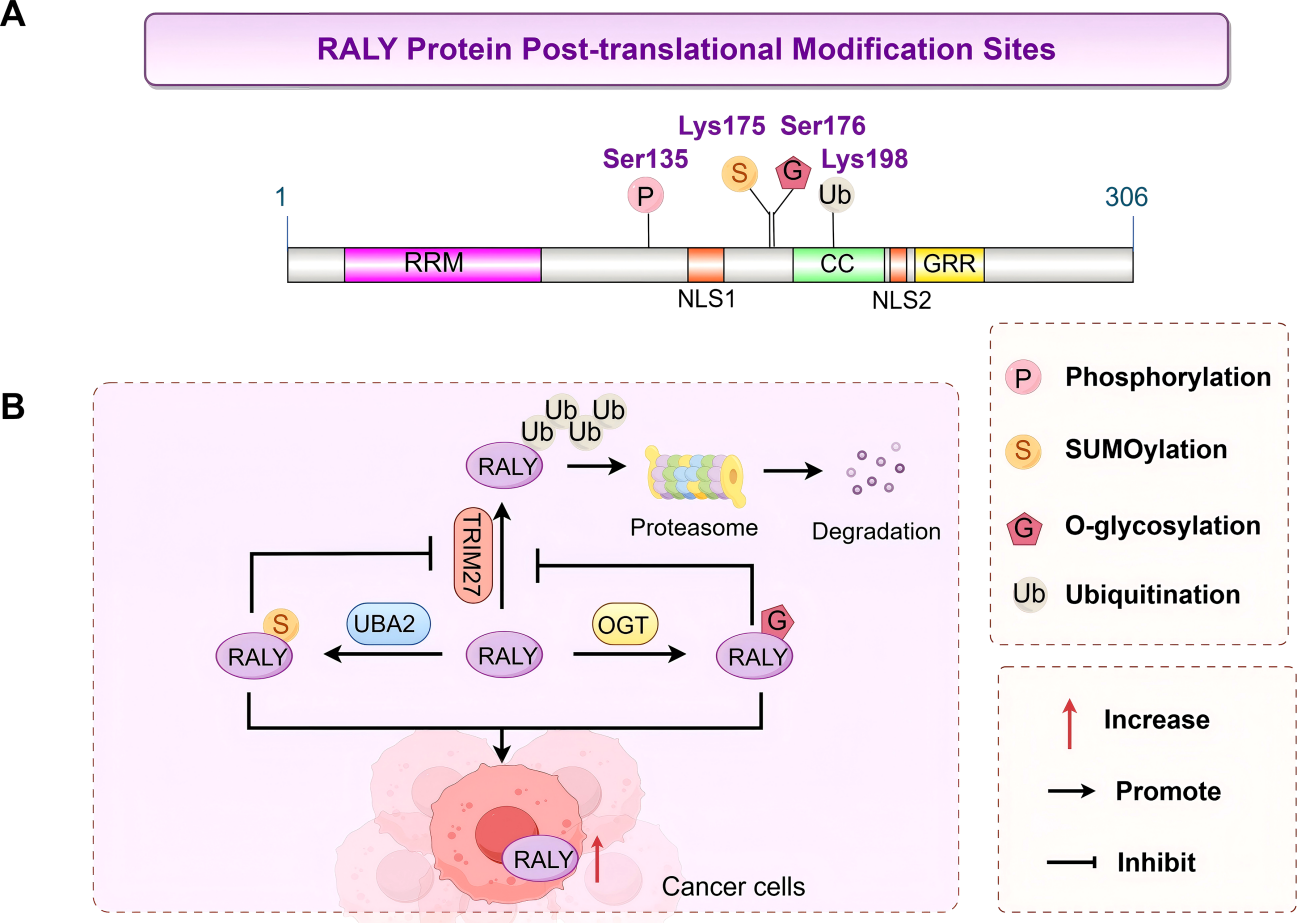
Post-translational modification sites and regulatory network of the RALY protein: **(A)** Distribution of post-translational modification sites of RALY. This panel shows the amino acid residues that undergo post-translational modifications on the RALY protein, including Ser135 (phosphorylation, labeled “P”), Lys175 (SUMOylation, labeled “S”), Ser176 (O-glycosylation, labeled “G”), and Lys198 (ubiquitination, labeled “Ub”). **(B)** Regulatory network of RALY post-translational modifications. UBA2 mediates the SUMOylation of RALY, while OGT catalyzes its O-glycosylation; both modifications can enhance the stability of the RALY protein by antagonizing the ubiquitin-dependent degradation pathway (e.g., the degradation initiated by TRIM27-mediated ubiquitination of RALY), thereby leading to its abnormal accumulation in cancer cells. By Figdraw.

Multiple studies have confirmed that RALY can undergo numerous PTMs at specific amino acid residue sites. These modifications include phosphorylation, ubiquitination, small ubiquitin-like modifier (SUMO) modification (SUMOylation), O-linked N-acetylglucosamine (O-GlcNAc) glycosylation, etc. (**Figure 2A**). They can dynamically regulate RALY’s subcellular distribution, protein stability, and capacity to bind to target RNAs and proteins and provide the molecular basis for RALY’s functional variety. Among the various PTMs of RALY, ubiquitination is the most extensively studied, and it exhibits distinct chain-type-dependent differences in exerting its functions. Research by Herrmann et al. has shown that RALY can synergistically bind to adenoviral RNA together with hnRNP C, thereby inhibiting viral late gene expression and the host immune response. In contrast, the early region 1B 55-kDa protein (E1B-55K)/early region 4 open reading frame 6 (E4orf6) complex can mediate non-degradative ubiquitination of RALY at lysine 198 (K198) with non-K48-linked chains; this modification reverses the aforementioned inhibitory effect and ultimately promotes viral replication (18). In addition, in Hep3B and Huh7 hepatocellular carcinoma cell lines, RALY can bind to the E3 ubiquitin ligase tripartite motif containing 27 (TRIM27), which induces RALY degradation through the ubiquitin-proteasome pathway to regulate RALY’s protein homeostasis even though the specific modification site remains unclear (24). Beyond ubiquitination, other PTMs, which include the phosphorylation modification of RALY at serine 135 (Ser135) mediated by Protein Kinase A (PKA), also play critical roles in the functional regulation of RALY. This modification is the key determinant that underpins the aberrant activation of RALY-induced signaling pathways associated with tyrosine kinase inhibitor (TKI) resistance in lung adenocarcinoma, such as the canonical epidermal growth factor receptor (EGFR)/phosphatidylinositol 3-kinase (PI3K)/protein kinase B (AKT) pathway (45). Both O-GlcNAc modification and SUMOylation boost the stability of RALY protein. They do this by blocking the ubiquitin-dependent degradation pathway. This action in turn causes RALY to build up abnormally in cancer cells. Finally, they exert oncogenic effects by regulating downstream signaling pathways. In hepatocellular carcinoma, O-GlcNAc transferase (OGT) catalyzes the O-GlcNAc modification of RALY at Ser176. This modification directly blocks RALY from binding to the E3 ubiquitin ligase TRIM27. As a result, it inhibits the degradation process that is mediated by the ubiquitin-proteasome pathway (24). In glioma, ubiquitin-like modifier-activating enzyme 2 (UBA2)-mediated SUMO1 modification of RALY at lysine 175 (Lys175) significantly improves its protein stability. The proteasome inhibitor MG132 can inhibit the degradation of RALY, a phenomenon that not only confirms that RALY degradation relies on the ubiquitin-proteasome pathway but also demonstrates that SUMO1 modification achieves the stability of RALY protein precisely by blocking this degradation pathway (26). In summary, the various PTMs of RALY do not function independently; instead, crosstalk between some modifications can form a complex regulatory network (46). Among these, the antagonistic effects between O-GlcNAc modification, SUMOylation, and ubiquitination constitute a key mechanism underlying the abnormal stability of RALY and the promotion of cancer progression in tumors (**Figure 2B**).

### 3.4 Synergistic integration of regulatory networks and future perspectives

The functional regulation of RALY does not rely on a single mechanism but instead constitutes a “multi-layered, synergistic” core regulatory network formed by m^6^A epigenetic modification, PPIs, and PTMs (47). To be specific, by changing the RNA structure, m^6^A modification indirectly regulates RALY’s capacity for RNA processing, while PPIs expands RALY’s functional scope via forming protein complexes. PTMs directly affect RALY’s protein stability and activity through dynamic changes. These three pathways work both on their own and together in a coordinated way. Together, they decide RALY’s final functional role. This not only helps us understand why RALY has different functions in physiological and pathological processes but also uncovers the key molecular mechanism behind its abnormal activity in tumors. Future studies should focus on exploring the crosstalk and molecular links among these three regulatory pathways. This work will improve our systematic understanding of the RALY regulatory network. It will also provide more precise and targeted theoretical support for developing RALY-targeted cancer treatment strategies.

## 4 Mechanisms and clinical significance of RALY in the digestive system and other systemic tumors

RALY is abnormally overexpressed in a variety of malignant tumors. Through diverse molecular regulatory mechanisms, it is extensively involved in malignant biological behaviors of tumor cells, including proliferation, migration, invasion, and chemoresistance. The underlying mechanisms of RALY action exhibit tumor specificity and complexity (**Table 2**).

**Table 2:** Mechanism of action and clinical significance of RALY in different tumors.

Cancer type	Dysregulation	Molecular mechanism	Role	Clinical significance	References
Hepatocellular carcinoma	Upregulated	RALY regulates USP22 mRNA nuclear export via O-GlcNAc modification; The RALY-SF3B3 complex cooperatively regulates alternative splicing of MTA1 pre-mRNA (↓MTA1-S, ↑MTA1-L), thereby activating the cholesterol synthesis pathway; Downregulates the expression of E-cadherin	Oncogenic	Associated with poor prognosis in patients	(24) (48) (51)
Colorectal cancer	Upregulated	RALY regulates miRNA (e.g., miR-483/676/877) processing via the “m^6^A switch” mechanism, reprogramming mitochondrial metabolism; Enhances PLD2 mRNA stability in an m^6^A-dependent manner, promoting exosome biogenesis and M2 polarization of macrophages	Oncogenic	Associated with poor prognosis in patients	(42) (54)
Pancreatic cancer	Upregulated	USP11 regulates the RALY/FXYD5 signaling axis via deubiquitination modification, thereby promoting aerobic glycolysis in pancreatic cancer	Oncogenic	—	(25)
Lung cancer	Upregulated	Activates the c-Myc-Cyclin D1/CDK4-p27 and Rho/MMP9/EMT signaling axes; Forms a RALY-USP7-MDM2 ternary complex via protein-protein interaction, promoting ubiquitin-mediated degradation of p53	Oncogenic	Associated with poor prognosis in patients	(27) (44)
Breast cancer	Upregulated	Regulates alternative splicing of PRMT1 pre-mRNA to generate the oncogenic isoform PRMT1v2	Oncogenic	Associated with poor prognosis in patients	(63)
Cervical cancer	Upregulated	Regulates cell cycle progression; Regulates alternative splicing of the FOS gene and transcription factor activity	Oncogenic Tumor-suppressive	—	(10) (23)

↓ indicates a decrease, and ↑ indicates an increase.

### 4.1 Hepatocellular carcinoma

**Figure 3:**
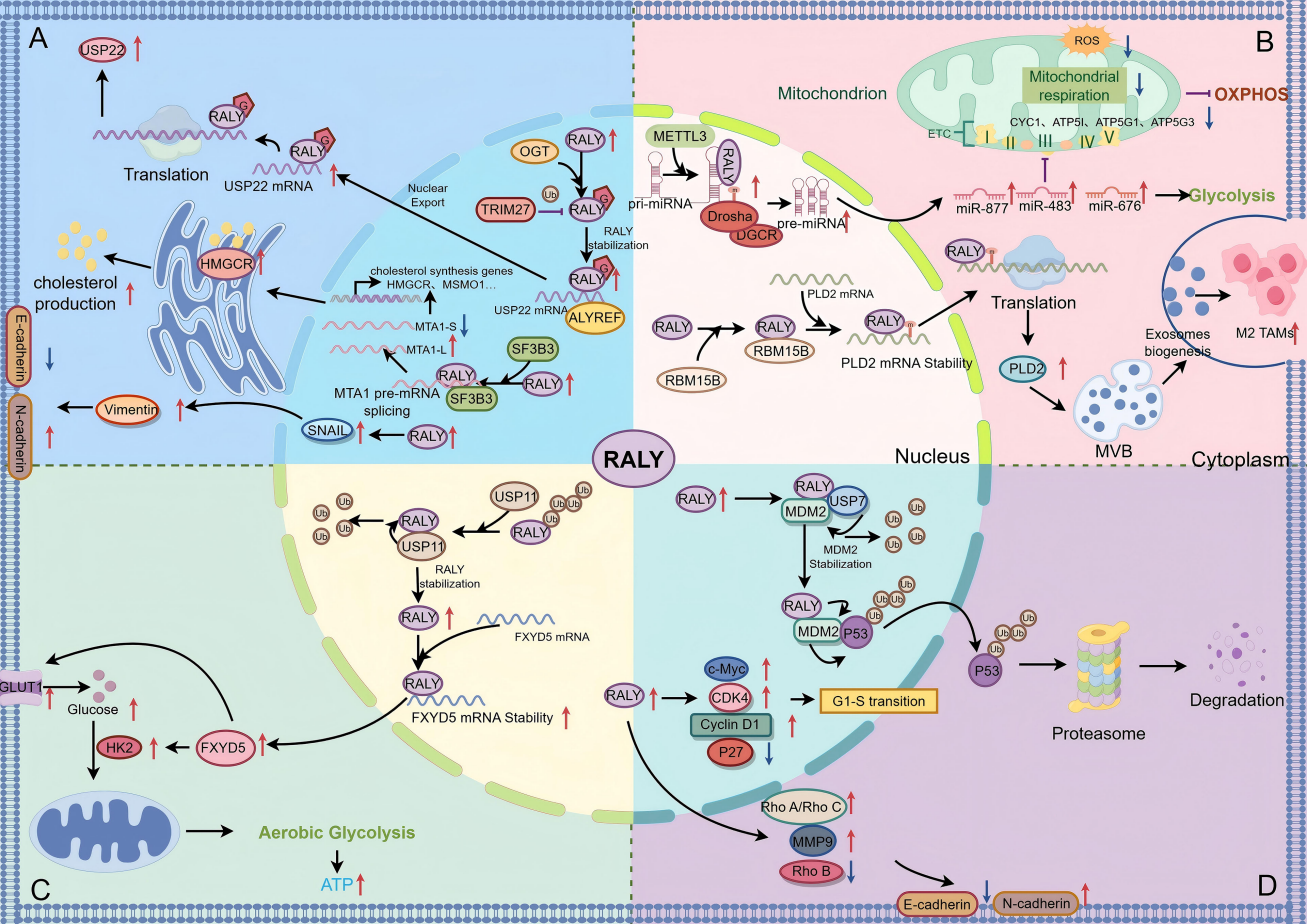
Schematic diagram of the functional regulatory mechanisms of RALY in different cancers. Red arrows (↑), increase; blue arrows (↓), decrease; black arrows, promotion; purple symbol (⊣), inhibition. G, O−GlcNAcylation; Ub, ubiquitination; m, m^6^A modification. **(A)** In hepatocellular carcinoma: Via O-GlcNAc modification, RALY regulates the nuclear export of USP22 mRNA; it also forms a complex with SF3B3 to modulate the alternative splicing of MTA1 pre-mRNA and regulates the expression of epithelial-mesenchymal transition-related proteins, thereby promoting the proliferation and metastasis of hepatocellular carcinoma cells. **(B)** In colorectal cancer: RALY regulates the processing and maturation of miRNAs (e.g., miR-483, miR-676, miR-877) through the “m^6^A switch” mechanism, thereby participating in the regulation of the expression of mitochondrial respiration (OXPHOS)-related molecules (e.g., CYC1, ATP5I, ATP5G1, ATP5G3) to achieve mitochondrial metabolic reprogramming. Meanwhile, it enhances the stability of PLD2 mRNA in an m^6^A-dependent manner, promoting exosome biogenesis and the polarization of macrophages toward the M2 phenotype, which ultimately drives the proliferation and metastasis of colorectal cancer cells. **(C)** In pancreatic cancer: RALY maintains its own protein stability via USP11-mediated deubiquitination, which in turn stabilizes the expression of FXYD5 mRNA, promotes aerobic glycolysis and ATP production, and provides energy support for the metabolic activities of cancer cells. **(D)** In lung cancer: RALY forms a ternary complex with USP7 and MDM2 to promote the ubiquitin-mediated degradation of p53; it also activates the c-Myc-Cyclin D1/CDK4-p27 axis and the Rho/MMP9/EMT axis, thereby driving the proliferation and metastasis of lung cancer cells. By Figdraw.

In recent years, there’s been notable progress in research on RALY in hepatocellular carcinoma (HCC), and many studies have shown the cancer-promoting effects of RALY in this disease. Clinical retrospective studies have confirmed that RALY is significantly highly expressed in HCC tissues, and its high expression is closely associated with tumor size, serum alpha-fetoprotein (AFP) levels, advanced tumor-node-metastasis (TNM) stage, and poor prognosis in patients. Subsequent mechanistic studies demonstrated that RALY exerts its oncogenic function by activating the cholesterol synthesis pathway through regulating metastasis-associated protein 1 (MTA1) alternative splicing. RALY forms a complex with splicing factor 3b subunit 3 (SF3B3) to regulate MTA1 splicing from the tumor-suppressive isoform (MTA1-S) to the oncogenic isoform (MTA1-L). Reduced MTA1-S relieves transcriptional inhibition of downstream cholesterol synthesis genes (e.g., 3-hydroxy-3-methylglutaryl-coenzyme A reductase (HMGCR), sterol regulatory element-binding protein 1 (SREBP1)), thereby activating cholesterol synthesis and supporting rapid HCC cell proliferation (48) (**Figure 3A**). O-GlcNAc modification, as a common abnormal epigenetic modification in tumors, has been confirmed to be involved in the initiation and progression of hepatocellular carcinoma (49, 50). Liu et al.’s study showed the key role of O-GlcNAc modification in regulating RALY function; specifically, OGT catalyzes the O-glycosylation of RALY, which blocks the ubiquitin-dependent degradation triggered by the E3 ubiquitin ligase TRIM27, enhances RALY protein stability, and then promotes the expression of ubiquitin-specific protease 22 (USP22) to speed up the proliferation of HCC cells (24) (**Figure 3A**). In addition, RALY can promote HCC metastasis by inducing the EMT process, which is specifically manifested by the downregulated expression of the epithelial marker E-cadherin, along with the upregulated expression of the mesenchymal markers N-cadherin and vimentin and the EMT transcription factor Snail (51) (**Figure 3A**). Notably, a recent study found that RALY acts as a hub gene in hepatitis B virus-related acute-on-chronic liver failure (HBV-ACLF), which suggests it may play a cross-stage regulatory role in the disease progression pathway of “hepatitis B - liver failure - liver cancer” (52).

### 4.2 Colorectal cancer

Colorectal cancer (CRC) is the third most common malignant tumor worldwide and the second leading cause of cancer-related deaths, posing a major threat to human health (53). The Cancer Genome Atlas (TCGA) database analysis reveals frequent gene amplification of RALY in CRC. Clinical cohort analysis (n=130) further confirms that RALY is significantly overexpressed in tumor tissues compared with adjacent normal controls, and its high expression correlates with advanced TNM stage, distant metastasis, and poor prognosis independent of KRAS proto-oncogene, GTPase (KRAS)/B-Raf proto-oncogene, serine/threonine kinase (BRAF) mutations and microsatellite instability (MSI) status. Notably, studies showed that RALY overexpression correlates with reduced expression of mitochondrial electron transport chain (ETC) genes like ATP synthase, H+ transporting, mitochondrial membrane subunit e (ATP5I) and ATP synthase, H+ transporting, mitochondrial Fo complex subunit C1 (ATP5G1), and this expression pattern ties to poor prognosis in CRC patients. At the mechanistic level, RALY acts as a key regulatory component of the Drosha complex and binds to methyltransferase-like 3 (METTL3)-mediated m^6^A-modified pri-miRNAs. By recruiting these pri-miRNAs to the Drosha-DiGeorge syndrome critical region 8 (DGCR8) complex, RALY facilitates the processing and maturation of miR-483, miR-676, and miR-877. Consequently, these mature miRNAs suppress mitochondrial oxidative phosphorylation (mtOXPHOS) in CRC cells by downregulating ATP5I, ATP5G1, ATP synthase, H+ transporting, mitochondrial Fo complex subunit C3 (ATP5G3), and cytochrome c1 (CYC1), ultimately promoting tumor cell proliferation (42) (**Figure 3B**). When it comes to how RALY regulates metastasis, it teams up with RNA-binding motif protein 15B (RBM15B) to directly bind phospholipase D2 (PLD2) mRNA in an m^6^A-dependent way, and this binding enhances PLD2 mRNA stability, increases PLD2 expression, and promotes multivesicular body formation and exosome secretion, which in turn induces macrophage M2 polarization and creates a tumor microenvironment that encourages metastasis (54) (**Figure 3B**). As a key enzyme in lipid metabolism, PLD2 hydrolyzes phosphatidylcholine to generate phosphatidic acid, which provides core signaling support for exosome biogenesis and secretion (55). On this basis, its follow-up studies further revealed that the natural active ingredient Astragaloside IV (ASIV) can block the biogenesis and release of tumor exosomes by targeting the RALY/PLD2 signaling axis, thereby significantly alleviating the metastasis of colorectal cancer (56). Notably, RALY is also associated with chemotherapy resistance in CRC. Specifically, RALY forms a complex with non-POU domain-containing octamer-binding protein (NONO) and Y-box binding protein-1 (YB-1) to affect chemotherapy resistance, and knockdown of RALY markedly enhances the sensitivity of CRC cells to oxaliplatin, which suggests that targeting RALY is a potential way to overcome oxaliplatin resistance in CRC (22). However, preclinical evidence only supports a close link between RALY and oxaliplatin resistance in CRC cells, whereas its clinical translational potential and relevance to patient chemotherapy response remain unvalidated and require further investigation.

### 4.3 Pancreatic cancer

Pancreatic cancer (PC) is one of the most aggressive cancer types of the digestive system, which is known as the “king of cancers” because of its extremely poor prognosis, strong aggressiveness, and rapid progression (57, 58). In recent years, studies have gradually revealed the oncogenic role and regulatory mechanisms of RALY in PC, providing new insights for molecularly targeted therapy of this disease. RALY is highly expressed in Panc-1 cell lines, and knocking down RALY can induce cell cycle arrest at the G1 phase, which points to its role in regulating PC cell proliferation (5). A recent systematic study further confirmed that knocking down RALY can greatly suppress the glycolytic phenotype of PC cells, as shown by reduced lactate production and ATP levels as well as downregulated expression of glucose transporter 1 (GLUT1) and hexokinase 2 (HK2). Mechanistically, ubiquitin-specific protease 11 (USP11) maintains RALY protein stability via deubiquitination modification; accumulated RALY then binds to the 3’UTR of FXYD domain-containing protein 5 (FXYD5, also called Dysadherin) mRNA to enhance its mRNA stability and increase protein expression (25) (**Figure 3C**). As a widely recognized positive regulator of aerobic glycolysis in tumors, FXYD5 boosts glucose uptake and glycolytic activity in tumor cells by activating the GLUT1/HK2 axis and ultimately driving aerobic glycolysis and the malignant progression of tumor cells (59). Basic research in Panc-1 cell lines has initially clarified the oncogenic function of RALY in PC via regulating aerobic glycolysis, while the relevant clinical evidence (e.g., correlation with clinicopathological parameters and patient prognosis) remains insufficient and awaits further validation in large patient cohorts.

### 4.4 Lung cancer

Lung cancer (LC) is the leading cause of cancer-related deaths worldwide, with non-small cell lung cancer (NSCLC) accounting for 85% of all cases (60). Clinical tissue analyses and survival analyses demonstrate that RALY is significantly overexpressed in NSCLC tissues and closely associated with lymph node metastasis and poor overall survival. In functional assays, silencing RALY suppresses the proliferation, migration, and invasion of NSCLC cells and induces G1-phase cell cycle arrest. Mechanistically, RALY depletion downregulates cellular myelocytomatosis oncogene (c-MYC), Cyclin D1, cyclin-dependent kinase 4 (CDK4), matrix metallopeptidase 9 (MMP9), Ras homolog family member A/C (RhoA/RhoC), N-cadherin, and β-catenin, while upregulating cyclin-dependent kinase inhibitor 1B (CDKN1B, p27), Ras homolog family member B (RhoB), and E-cadherin. These changes collectively indicate that RALY promotes NSCLC progression by regulating cell cycle and EMT processes via the c-Myc-Cyclin D1/CDK4-p27 axis and the Rho/MMP9/EMT axis (27) (**Figure 3D**). In addition, Hu et al. reported that RALY can form a ternary complex with USP7 and MDM2, enhancing the deubiquitination stabilization effect of USP7 on MDM2. The stabilized MDM2 further promotes the ubiquitination and degradation of p53, which further drives tumorigenesis by inhibiting the p53 tumor-suppressive pathway (44, 61) (**Figure 3D**). In summary, through the multi-dimensional synergistic mechanisms of “driving cell cycle progression, activating EMT, and inhibiting the p53 pathway,” RALY is deeply involved in the proliferation, invasion, and metastasis of NSCLC and is directly associated with poor prognosis in patients, thus holding promise as a novel prognostic biomarker and therapeutic molecular target.

### 4.5 Breast cancer

Breast cancer (BC) is the malignant tumor with the highest incidence and mortality rates among women worldwide, and it exhibits high heterogeneity. Based on molecular markers, it can be classified into subtypes including Luminal A, Luminal B, human epidermal growth factor receptor 2 (HER2)-positive, and triple-negative breast cancer (TNBC), among which tumor metastasis is the key cause of poor prognosis in patients (62). In recent years, the regulatory role of RALY in breast cancer metastasis has been gradually uncovered, providing a novel molecular target for precise diagnosis and treatment of the disease. Bondy-Chorney et al. (63) found that RALY expression in breast cancer tissues is significantly higher than that in adjacent normal tissues; patients with high RALY expression have markedly shortened overall survival, and this correlation is more pronounced in the estrogen receptor-positive/HER2-negative (ER+/HER2−) subtype, indicating that RALY is a potential biomarker for poor prognosis in breast cancer. Mechanistically, RALY can directly bind to the pre-mRNA of protein arginine methyltransferase 1 (PRMT1), facilitate the selective inclusion of exon 2 to generate the cancer-promoting isoform PRMT1v2, and thereby drive breast cancer metastasis. Knocking down RALY reduced cancer cell invasion considerably, but exogenous PRMT1v2 replenishment reversed this effect. However, the regulatory network that controls PRMT1 pre-mRNA alternative splicing is highly complex, and experimental evidence on whether RALY cooperates with other splicing factors to precisely regulate this process is still lacking (64); to fill this research gap, future studies should explore RALY’s downstream molecular mechanisms in breast cancer and strengthen the theoretical basis of its cancer-promoting pathway.

### 4.6 Other cancers

RALY has also been shown to participate in the initiation and progression of many other malignant tumors, such as ovarian cancer (OC), bladder cancer, cervical cancer (CC), glioma, multiple myeloma (MM), and melanoma (22). In cervical cancer research, both RALY mRNA and protein levels are significantly upregulated in HeLa cells. Silencing RALY causes G1−phase arrest and inhibits cell proliferation, supporting an oncogenic role of RALY (10). However, a recent study reported that RALY overexpression suppresses cervical cancer cell proliferation and promotes apoptosis. This regulatory effect is associated with RALY-mediated alternative splicing of FOS and transcription factor activity (23). These conflicting findings imply a context-dependent dual role of RALY in cervical cancer. The contradictory functions of RALY may arise from several key factors. First, cervical cancer includes etiologically distinct subgroups: human papillomavirus (HPV)-dependent and HPV-independent tumors. Persistent high-risk HPV infection (as in HeLa cells) continuously disrupts cell cycle and apoptotic pathways, which may fundamentally change the downstream regulatory functions of RALY. Second, tumors have inherent genetic heterogeneity, such as different mutation statuses of oncogenes and tumor suppressors (e.g., tumor protein p53 (TP53) and retinoblastoma 1 (RB1)) and varying activation of core signaling pathways. This heterogeneity can lead to different functional phenotypes even for the same gene (65). Third, estrogen receptor signaling may crosstalk with RNA-binding proteins, and differences in estrogen levels or receptor expression may further modify the function of RALY (66). Finally, RALY may regulate distinct downstream target genes and alternative splicing networks in a context-specific manner, which also contributes to its paradoxical roles. Notably, all the above contradictory conclusions are based on *in vitro* studies using cervical cancer cell lines. At present, no clinical data are available for RALY in cervical cancer. Its expression profile in clinical tumor tissues, association with HPV subtype or estrogen receptor status, and clinical prognostic value still require validation in large-sample clinical cohorts. Therefore, further combined basic and clinical studies are needed to confirm and clarify these opposing effects. In the field of precancerous lesions, RALY is specifically upregulated in lithocholic acid-treated CP-A cells of Barrett’s esophagus. As a molecule associated with precancerous lesions of esophageal adenocarcinoma, RALY holds promise as a potential biomarker for assessing disease progression risk, thereby providing a novel target for the early clinical identification of high-risk lesions (67). However, this study has not yet verified its expression characteristics or correlation with clinical parameters in precancerous lesion tissues, and future investigations should combine clinical samples and functional experiments to clarify its translational value. Overall, except for the conflicting roles reported in cervical cancer, most studies have confirmed that RALY can promote the proliferation, migration, and invasion of tumor cells.

### 4.7 The regulatory mechanisms of RALY in different tumors: common features and tissue-specific differences

Based on the above research progress of RALY in different tumors, it can be seen that although its regulatory mechanism is partially overlapped, its downstream signaling pathway shows significant differentiation characteristics due to tumor tissue specificity. From the perspective of common mechanism, RALY mainly plays a role in promoting cancer through three core ways: one is to regulate the alternative splicing of target genes (such as MTA1 in HCC, PRMT1 in breast cancer and FOS in cervical cancer), which is the core and most extensive regulation mode as RBP; the second is to participate in the regulation of protein PTMs, and maintain protein stability by affecting self-ubiquitination (TRIM27-mediated in HCC, USP11-mediated in PC) or glycosylation (OGT-catalyzed in HCC), thereby enhancing cancer-promoting function; the third is to mediate tumor metabolic reprogramming, participate in the regulation of key metabolic pathways such as cholesterol synthesis, glycolysis, and mtOXPHOS, and provide a material and energy basis for malignant proliferation of tumor cells.

From the perspective of tissue-specific differences, the regulation of RALY in digestive system tumors is centered on metabolic reprogramming: HCC is driven by abnormal activation of the cholesterol synthesis pathway, PC relies on the GLUT1/HK2 axis to up-regulate glycolysis activity, and CRC inhibits mtOXPHOS by regulating miRNA maturation, which is highly consistent with the pathological characteristics of digestive system tumors that are active in metabolism and rely on rapid nutrient intake. In other system tumors, the regulation of RALY is more biased towards the classical cancer pathway and EMT process: lung cancer inhibits the tumor suppressor pathway through the MDM2/USP7/p53 axis, while activating the Rho/MMP9/EMT axis to promote metastasis; PRMT1 alternative splicing is the core driver of metastasis in BC, and the functional correlation is more significant in ER+/HER2− subtypes.

In summary, RALY, as a functionally heterogeneous oncogene, is involved in the occurrence and development of various tumors. It is worth noting that its regulatory mechanisms have both common characteristics and significant tissue specificity in different tumors. RALY mainly promotes tumor development by regulating RNA splicing, protein modification, and metabolic reprogramming. However, its dual role in cervical cancer remains unclear and needs further study. In addition, the functions and mechanisms of RALY in many other tumors have not been fully explored and also require further investigation. Therefore, more research on RALY will help clarify cancer mechanisms and provide a theoretical basis for new targeted therapies.

## 5 RALY-targeted tumor therapeutic strategies: advances, challenges and translational prospects

Based on the core cancer-promoting role of RALY in tumorigenesis and development, the treatment strategy targeting this protein has become a research hotspot in the field of tumor precision therapy, with substantial potential for clinical translation.

### 5.1 Strategic classification and research advances

At present, the core strategies of related research are mainly divided into two categories: direct targeted intervention and combined treatment. In terms of direct intervention, small interfering RNA (siRNA) or short hairpin RNA (shRNA) can inhibit the malignant phenotype of tumors by specifically knocking down the expression level of RALY. This strategy has been validated in patient-derived xenotransplantation (PDX) and patient-derived organoid (PDO) models of colorectal cancer, which can significantly block tumor progression and lay a solid foundation for subsequent clinical transformation (42, 68). In addition, peptide-based proteolysis-targeting chimeras (p-PROTACs) have the characteristics of high specificity and low cytotoxicity due to the greater advantages of the interaction surface between peptide ligands and target proteins (69, 70). The RALY-p-PROTAC molecule designed by Liu et al. can specifically degrade RALY protein in a hepatocellular carcinoma model and effectively inhibit the proliferation of hepatocellular carcinoma cells (24). This work provides a novel paradigm for developing small-molecule drugs targeting RALY. Regarding combination therapy, functional studies confirm that RALY knockdown can significantly enhance the killing effect of traditional chemotherapeutic drugs (e.g., oxaliplatin and 5-fluorouracil) on colorectal cancer cells, indicating that RALY may serve as a promising chemosensitization target for combination regimens (22, 71). It is worth noting that all current RALY-targeted strategies are still in the preclinical development stage, and their *in vivo* efficacy, safety, and targeting still need to be further verified to promote their clinical translation (72, 73).

### 5.2 Current challenges and future translational prospects

Despite encouraging progress in preclinical studies, the clinical translation of RALY-targeted therapies faces three major challenges. First, drug delivery efficiency is insufficient: nucleic acid drugs represented by siRNA show poor *in vivo* stability, are easily degraded by nucleases, and display limited tumor penetration and accumulation (72). Second, target specificity is not ideal: new agents such as p-PROTACs still carry off-target risks, which could harm normal organs that highly express RALY (73). Third, tumor heterogeneity limits broad applicability: RALY regulatory networks differ substantially across tumor types and even within subtypes, limiting the efficacy of single-target strategies.

To address these current challenges and accelerate clinical translation, future studies should focus on the following improvements: First, clarify the specific regulatory network of RALY in different tumor types to support precise targeting; Second, optimize delivery systems, including viral vectors and lipid nanoparticles, to improve tumor targeting and stability *in vivo*; Third, develop new tools such as splicing-switching antisense oligonucleotides (ASOs) (74) and allosteric inhibitors targeting the RALY complex in order to enrich targeting methods and reduce off-target effects. With continuous technological optimization, the clinical translation of RALY-centered targeted therapies is expected to be further accelerated.

## 6 Summary and outlook

As a key member of the hnRNP family, RALY exerts its core function through RNA-specific binding. Via multiple molecular mechanisms, including regulating the alternative splicing of target RNAs, mediating its own PTMs, and driving tumor metabolic reprogramming, RALY plays a critical oncogenic role in most malignant tumors such as hepatocellular carcinoma, colorectal cancer, lung cancer, and breast cancer. Retrospective tissue-based studies and public database analyses have demonstrated that abnormally high expression of RALY is closely related to malignant tumor progression and poor patient prognosis, suggesting its potential value as a tissue prognostic marker. Furthermore, the functional regulatory network of RALY involves complex crosstalk of m^6^A modification, PPIs, and PTMs (ubiquitination, O-GlcNAcylation, etc.) and exhibits significant tumor heterogeneity, which constitutes the core molecular basis for its involvement in tumorigenesis and progression.

Based on the current research status and existing scientific issues, future studies can focus on three directions to deepen mechanistic understanding and promote clinical translation: First, precision of mechanistic dissection: integrating technical approaches including high-throughput sequencing, single-cell multi-omics, proteomics, and metabolomics to systematically characterize the patterns of abnormal expression and functional heterogeneity of RALY across different tumor subtypes and precancerous lesions, clarify its tissue-specific regulatory targets as well as the interaction mechanisms with the tumor microenvironment and immune cells, and thereby refine the regulatory chain of structure-function-pathological phenotype. Second, innovative development of translational tools: relying on bioinformatics, structural biology, and medicinal chemistry techniques to thoroughly characterize the interaction interface between RALY and its target RNAs/proteins; developing highly efficient and specific targeted intervention tools (e.g., small-molecule allosteric inhibitors, targeted siRNAs, peptide-based PROTACs, and CRISPR/Cas9 gene-editing technologies) (75). Third, standardization and clinical validation of biomarkers: The biomarker value of RALY is currently limited to the tissue prognostic level, lacking a serum/body fluid detection system, and no core diagnostic efficacy indicators such as sensitivity, specificity, area under the curve (AUC), positive predictive value (PPV), and negative predictive value (NPV) have been reported. Large-scale clinical cohort studies are urgently needed to systematically evaluate the diagnostic and prognostic value of RALY alone or in combination with classic markers (AFP, carcinoembryonic antigen (CEA), carbohydrate antigen 19-9 (CA19-9), etc.), clarify its incremental value through head-to-head comparisons, establish standardized detection procedures and cut-off values, and promote its translation from basic research to clinical application.

With the deepening understanding of the molecular regulatory network and pathological functions of RALY, as well as the gradual improvement of targeted intervention technologies and clinical translation systems, RALY shows potential basic research value and preliminary translational prospects in precision oncology. Strategies targeting RALY can provide a theoretical basis and experimental foundation for overcoming tumor chemoresistance and exploring novel therapeutic approaches for advanced tumors. Under strictly defined application scenarios, RALY can serve as a prognostic reference indicator at the tissue level. Through large−sample clinical validation and standardized diagnostic efficacy evaluation in the future, it is expected to gradually transform into a clinically applicable biomarker and therapeutic target, ultimately providing new directions for optimizing individualized tumor diagnosis and treatment and improving patient prognosis.
